# A14 THE INTESTINAL MICROBIOTA CONTRIBUTES TO AGE-RELATED MEMORY DECLINE

**DOI:** 10.1093/jcag/gwab049.013

**Published:** 2022-02-21

**Authors:** N Kraimi, G De Palma, J Lu, D Bowdish, E Verdu, E Sibille, T Prevot, S M Collins, P Bercik

**Affiliations:** 1 Medicine, McMaster University, Hamilton, ON, Canada; 2 McMaster University Department of Pathology and Molecular Medicine, Hamilton, ON, Canada; 3 Centre for Addiction and Mental Health, Toronto, ON, Canada

## Abstract

**Background:**

Age-related deterioration of cognitive function and memory capacity occur in both humans and rodents. For example, significant memory deficits have been reported in conventionally raised (SPF) old mice compared to conventionally raised young mice submitted to a spatial memory task (Prevot *et al*., 2019, Mol Neuropsychiatry 5, 84–97). Microbiota-to-brain signaling is now well established in mice and humans, but the extent to which it influences age-associated memory decline is unknown.

**Aims:**

Our study examines whether the intestinal microbiota contributes to age-associated changes in brain function. We address the specific hypothesis that age-associated cognitive decline is attenuated in the absence of the intestinal microbiota.

**Methods:**

We assessed anxiety-like and depressive-like behavior, locomotor activity and spatial memory performance in young germ-free (GF) mice (2–3 months of age, n=24) and senescent GF mice (13–27 months old, n=22) maintained in axenic conditions, and compared them to conventionally raised (SPF) mice of the same age. Anxiety-like behavior, locomotor activity and depressive-like behavior were measured using the light-dark preference, open-field, and tail suspension tests. We also used the Y-maze test based on a spontaneous alternation task to assess cognition, with the alternation rate as a proxy of spatial working memory. The age-associated inflammation was assessed with IL-6 cytokine plasma concentrations measured by ELISA.

**Results:**

Anxiety-like behavior and depressive-like behavior did not change with the age regardless of the microbial status. However, old SPF mice traveled less distance (866.8 cm) than young SPF mice (1375 cm, *p* < 0.01) in the open-field. Similarly, old GF mice also traveled less distance (458.9 cm) than young GF mice (875.7 cm, *p* < 0.0001). In contrast to old SPF mice, old GF mice did not show memory impairment in the spatial memory task. Indeed, old SPF mice displayed lower alternation rate of 58.3%, compared to that found in young SPF mice (76.9%, *p* < 0.05) while both old and young GF mice had an identical alternation rate of 73.3% ( *p* > 0.05). In addition, IL-6 plasma levels revealed that old GF mice did not show signs of age-associated inflammation that was evident in old SPF mice (3.68 vs. 13.93 pg/ml, *p* < 0.05).

**Conclusions:**

We conclude that the absence of age-related memory deficit in old germ-free mice is consistent with a role for the microbiota in age-related cognitive decline, likely mediated via the immune system, as suggested by the absence of age-associated inflammation in germ-free mice. We propose that novel microbiota-targeted therapeutic strategies may prevent or delay the cognitive decline of aging.

**Funding Agencies:**

CIHRBalsam Family Foundation

